# Chronic abdominal distention caused by diffuse nodular ileal and mesenteric lipomatosis: A case report

**DOI:** 10.1097/MD.0000000000039171

**Published:** 2024-08-02

**Authors:** Li-xiao Zhang, Yong-fei Wang, Xiao-jie Hu, Jie Qi, Wei Liang

**Affiliations:** a Department of Gastrointestinal Surgery, Hebei General Hospital, Shijiazhuang, Hebei, China; b Department of Gynecology, Hebei General Hospital, Shijiazhuang, Hebei, China.

**Keywords:** case report, chronic abdominal distention, ileum, intestinal wall muscle layer, lipomatosis

## Abstract

**Rationale:**

Diffuse intestinal and mesenteric lipomatosis is a rare condition characterized by the overgrowth of adipose tissue in the intestines and mesentery. This case report aims to highlight the rare occurrence of chronic abdominal distention caused by this disease and its unique invasion into the muscle layer, which has not been previously reported.

**Patient concerns:**

A 36-year-old woman with a 7-year history of abdominal distension was admitted to our hospital’s Department of Gastrointestinal Surgery.

**Diagnose:**

Abdominal and pelvic computed tomography revealed diffuse small intestinal lipomatosis.

**Interventions:**

The patient underwent surgery. We performed an open-field ilectomy involving removal of all lipomatous intestines (250 cm).

**Outcomes:**

During the surgery, diffuse nodular ileal and mesenteric lipomatosis was confirmed, characterized by the presence of multiple nodular lipomas within the submucosal and muscular layers. The surgical intervention involved the resection of 250 cm of the affected ileum, followed by jejunoileal anastomosis. Postoperative pathology confirmed the diagnosis, with lesions observed in both the submucosa and muscle layers. The patient showed significant improvement in symptoms, with normal intestinal function and weight gain observed over a 10-month follow-up period, and no signs of recurrence.

**Lessons:**

Diffuse intestinal and mesenteric lipomatosis can lead to long-term abdominal distension. Additionally, it may be involved in the muscle layer of the intestinal wall. Surgery is the primary treatment option for symptomatic intestinal lipomatosis.

## 1. Introduction

Intestinal lipomatosis is a rare condition with unclear definition. Some definitions describe it as diffuse overgrowth and infiltration of well-differentiated adipose tissue in the submucosa of the small and large intestines,^[1]^ whereas others define it as the presence of more than 4 subcapsular lipomas.^[2]^ Although intestinal lipomatosis is typically asymptomatic, some patients may present with intermittent obstruction, colonic perforation, intussusception, or gastrointestinal bleeding.^[3–5]^ Additionally, intestinal lipomatosis can lead to abdominal distention owing to intermittent obstruction and altered bowel transport times. To date, no case of chronic abdominal distension caused by diffuse intestinal lipomatosis has been reported. This report presents a rare case of chronic abdominal distention resulting from diffuse nodular ileal lipomatosis and mesenteric lipomatosis in a 36-year-old woman.

## 2. Case presentation

**Figure 1.**
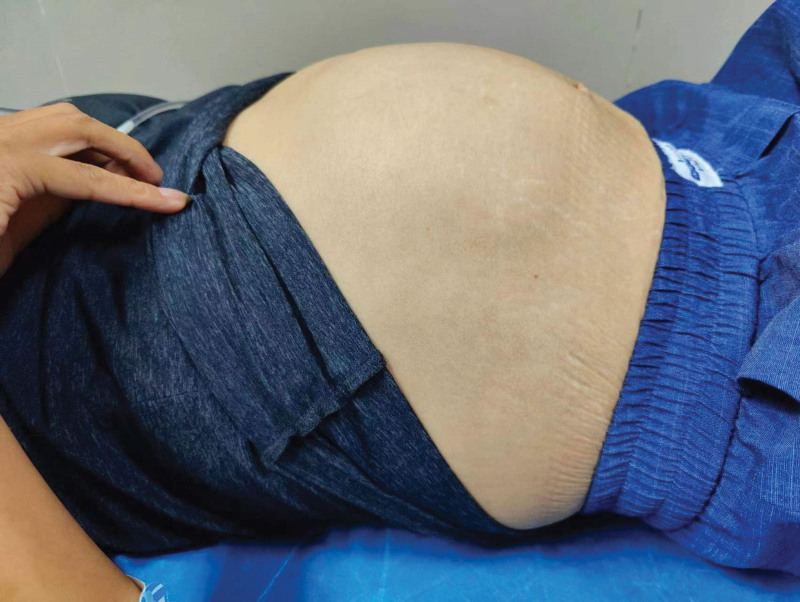
Physical examination showed significant abdominal distention.

**Figure 2.**
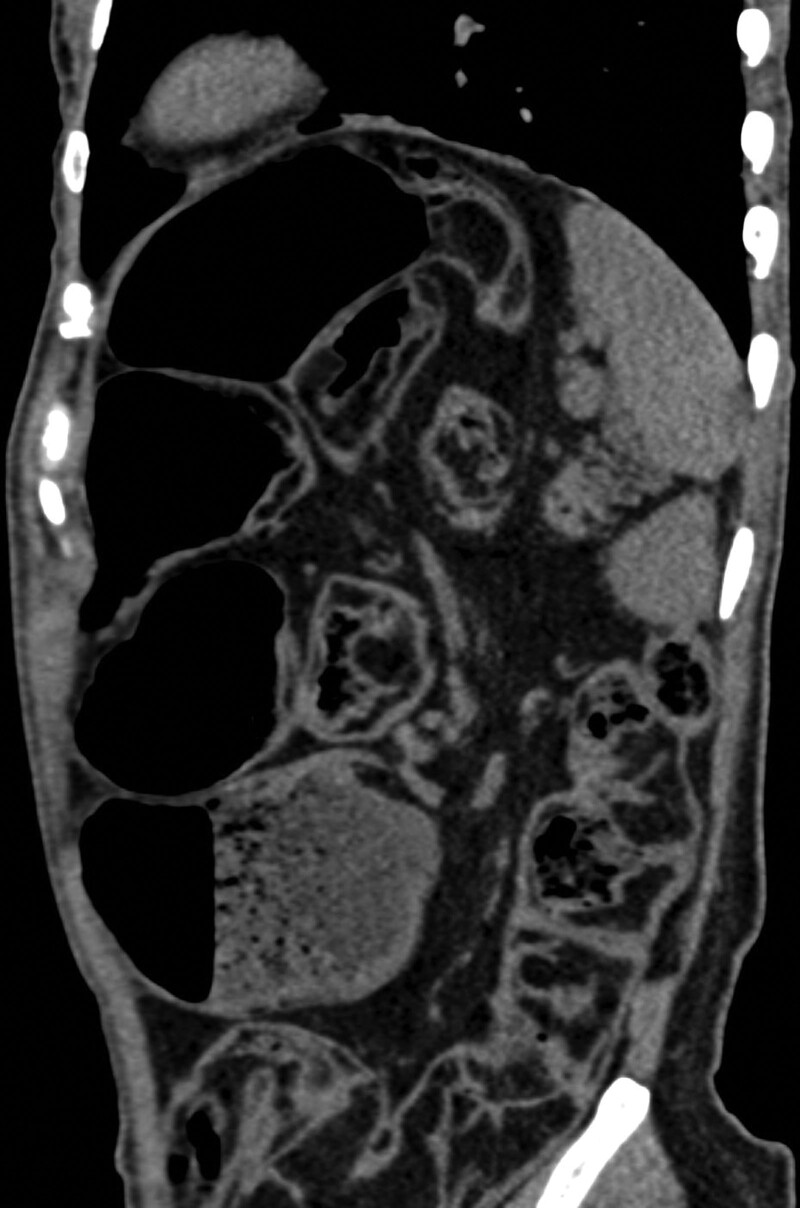
Coronary CT showed diffuse nodular submucosal lipomatous changes and significant intestinal dilatation.

**Figure 3.**
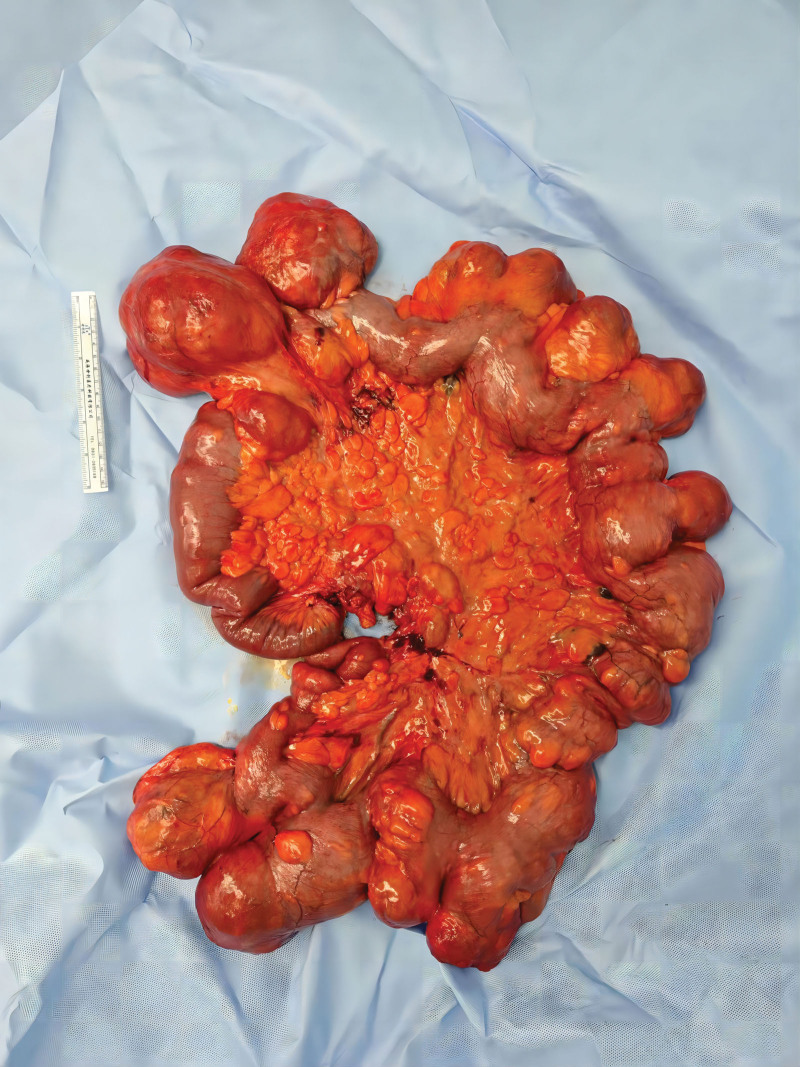
The postoperative gross specimen showed that most of the ileum was obviously enlarged and dilated, about 250 cm in length, with diffuse yellow adipose tissue deposition, but no obvious stenosis. In addition, multiple diffuse fat nodules 1 to 5 cm in diameter were found within the mesentery.

**Figure 4.**
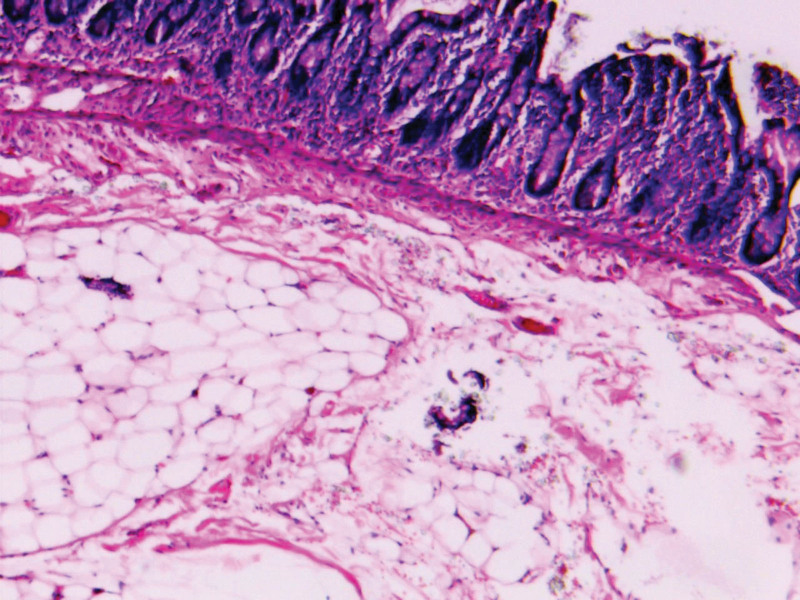
Postoperative pathology revealed multiple nodular lipomas in the submucosa and intestinal wall muscle layer, with no significant abnormalities in the mucosa.

A 36-year-old woman with a 7-year history of abdominal distension was admitted to the authors’ hospital. On physical examination, the abdomen appeared significantly distended (Fig. 1), but was non-tender upon palpation and exhibited normal bowel sounds. Her body mass index (BMI) was 18.31 kg/m^2^. Digital rectal examination revealed the absence of stool in the vault. Laboratory tests revealed a hemoglobin count of 108.00 g/L and an albumin level of 37.6 g/L. Computed tomography (CT) of the abdomen and pelvis (Fig. 2) revealed a partial accumulation of submucosal fat in the small bowel and significant intestinal dilatation. Intraoperatively, we observed that most of the ileum was significantly enlarged and dilated, measuring approximately 250 cm in length, with diffuse deposits of yellow fatty tissue but no significant stenosis. Multiple diffuse nodules, 1 to 5 cm in diameter were found within the mesentery (Fig. 3). Owing to the patient’s young age and healthy appearance of the intestine outside the lesion without signs of ischemia, we performed an open-field ilectomy involving the removal of all lipomatous intestines (250 cm), followed by anastomosis between the remaining jejunum (230 cm) and the distal ileum (20 cm). Postoperative pathology (Fig. 4) revealed multiple lipomas in the submucosa and intestinal wall muscle layer with no significant abnormalities in the mucosa. The patient was discharged 1 week after surgery. After 10 months of follow-up, her intestinal function returned to normal and she regained weight.

## 3. Discussion

Intestinal lipomatosis is morphologically classified into 4 categories: isolated lipoma, multiple circumscribed lipomas, diffuse nodular lipomatosis, and diffuse adipose tissue infiltration of the submucosa without tumor formation.^[6]^ The incidence of isolated lipomas at autopsy ranges from 0.04% to 4.5%.^[7]^ Multiple lipomas are found in approximately 5% of lipoma cases.^[1]^ Diffuse intestinal lipomatosis is a rare condition. Based on CT findings and postoperative pathology, the patient was diagnosed with diffuse nodular lipomatosis. Intestinal lipomatosis does not show any gender orientation, and the mean age of onset is 47.3 ± 18.4 years.^[3,8]^ Lesions of intestinal lipomatosis are typically confined to the submucosa but may also extend to the serosa and mesenteric fat.^[1]^ In this case, postoperative pathology revealed that adiposopathy not only infiltrated the lower mucosal layer of the intestinal wall, but also extended to the muscle layer, which is a surprising and significant finding that explains the rarity of this case.

Although several etiological factors have been suggested, the pathogenesis of intestinal lipomatosis remains unclear. These factors include embryo transfer of adipose tissue, congenital predisposition, degenerative diseases associated with adipometabolic disorders, post-chemotherapy fat deposition, chronic inflammatory diseases, infection, hamartoma syndrome, and gastric lipomatosis.^[9]^ Notably, the patient had no family or personal history of any of these conditions.

Intestinal lipomatosis is typically asymptomatic; however, some patients may experience abdominal pain, nausea, melena, diarrhea, constipation, intermittent vomiting, palpable masses, and weight loss.^[10]^ In this case, the main clinical symptom was long-term chronic abdominal distension, which was relatively rare. The coexistence of intestinal lipomatosis and diverticulosis has been previously reported, and is attributed to adipose infiltration and weakening of the intestinal wall. Diverticulosis can lead to bacterial overgrowth in the intestine, potentially causing malabsorption.^[11]^ The patient described in this report had nearly a dozen small intestinal diverticula of various sizes. Thus, the malnutrition in this patient may have been a consequence of malabsorption. During the 3-month follow-up, we observed a gradual weight gain in the patient.

CT is an excellent imaging modality for evaluating intestinal lipomatosis.^[12,13]^ In this case, abdominal pelvic CT revealed an extensive, infiltrative, nodular fatty mass with a density similar to that of normal adipose tissue, and a markedly dilated intestine. The location of the lesion in the ileum was confirmed by barium imaging.

If a patient presents with asymptomatic intestinal lipomatosis, specific treatment is not required. Endoscopic removal may be considered for lipomas smaller than 2.5 cm.^[14]^ For larger lipomas (>2.5 cm), local open excision and plastic repair^[15]^ or limited resection of the intestine containing the highest concentration of lipomas may be performed.^[14]^ Surgical resection is the only treatment option available for symptomatic diffuse fatty infiltration of the submucosal layer.^[15,16]^ Preservation of at least 180 cm of the intestinal loops during surgical resection is crucial to prevent short bowel syndrome.^[17]^ Preoperative assessment is particularly important in determining the extent of intestinal lipomatosis. In the present case, a combination of CT and barium enterography was used to diagnose diffuse submucosal lipomatosis of the ileum. Considering the patient’s young age and preserved quality of life, resection of the affected ileum was performed, followed by side-to-side jejunoileal anastomosis.

## 4. Limitations

This study is limited by its single case report nature, which may not be representative of the broader population affected by diffuse intestinal and mesenteric lipomatosis. Additionally, the long-term outcomes and recurrence rates of the disease remain unclear due to the limited follow-up period.

## 5. Conclusions

We present a rare case of diffuse nodular intestinal and mesenteric lipomatosis leading to chronic abdominal distention in a 36-year-old woman, in whom infiltration of the disease into the muscular layer of the intestinal wall was observed. Given its rarity, this condition may be easily overlooked during diagnosis. This possibility should be considered in the treatment of patients with chronic abdominal distension. CT of the abdominal cavity can provide definitive diagnosis. As demonstrated in this case, resection of the affected segment of the intestine remains the treatment of choice in patients with complications.

## Acknowledgments

We thank the patient for providing consent for publication of this article. We also thank Editage (www.editage.com) for the English language editing.

## Author contributions

**Writing – original draft:** Li-xiao Zhang.

**Data curation:** Yong-fei Wang, Xiao-jie Hu.

**Software:** Jie Qi.

**Writing – review & editing:** Wei Liang.
